# Chronic Cerebral Deterioration - A Comprehensive View of Old-Age Cerebral Deterioration

**DOI:** 10.14336/AD.2024.1073

**Published:** 2025-12-10

**Authors:** Jan Willem van Dalen

**Affiliations:** ^1^Department of Neurology, Donders Institute for Brain, Cognition, and Behaviour, Radboud University Medical Center, Nijmegen, the Netherlands.; ^2^Department of Public & Occupational Health, Amsterdam UMC, University of Amsterdam, Amsterdam, the Netherlands.

**Keywords:** Dementia, Alzheimer’s disease;, ageing, cognitive decline, neuropsychiatric symptoms, motor symptoms

## Abstract

The current one-dimensional view of pathological brain changes in older persons leading to cognitive complaints, mild cognitive impairment, and ultimately dementia is incomplete. It neglects the earliest, non-cognitive, and multifaceted symptoms of gradually accumulating cerebral damage. Subtle personality changes, balance problems, muscle wasting, weight loss, changing sleep patterns and declining blood pressure and cholesterol, precede memory problems and cognitive impairment. Chronic cerebral deterioration offers a new comprehensive concept, capturing symptoms across late-life cerebral dysfunction domains, and revising alleged dementia ‘risk factors’ more realistically into prodromal signs of cerebral deterioration. This may reduce research waste on dementia prevention unsuccessfully targeting prodromes and help identify people at the highest risk of developing care needs. It will improve counselling of older people with signs and symptoms when memory or other cognitive impairments are not yet present. Emphasizing total cerebral function over cognition alone focuses on what is clinically most relevant: the patient’s need of care.

## INTRODUCTION

Old-age dementia research mostly focusses on Alzheimer’s disease (AD): a syndrome of gradual cognitive decline, with memory loss as a core symptom, and specific AD protein depositions in the brain. In practice, the presentation of old-age dementia varies wildly. Memory complaints may be less prominent than other cognitive disturbances or neuropsychiatric symptoms including apathy, depression, hallucinations and delusions. Unexplained motoric and other somatic symptoms may occur, which are not considered core parts of dementia. Mounting evidence indicates that the current view of old-age dementia misses the bigger picture. It neglects the earliest, non-cognitive, and multifaceted symptoms of gradually accumulating cerebral damage. This has major detrimental consequences for both research and clinical practice.

### Current view

The Diagnostic and Statistical Manual of Mental Disorders (DSM-V) defines dementia as a major neurocognitive disorder, possibly preceded by a prodromal syndrome termed mild cognitive impairment (MCI) [1]. Both require objectified cognitive impairment, with restricted activities of daily living (ADL) in case of dementia. Cognitive domains include attention, praxis, language, social cognition, executive ability, learning and memory. Other recent concepts define AD as six stages based on AD biomarkers as ‘preclinical’ (stage 1-2), MCI (stage 3), or dementia (stages 4-6) [2].

**Figure 1.**
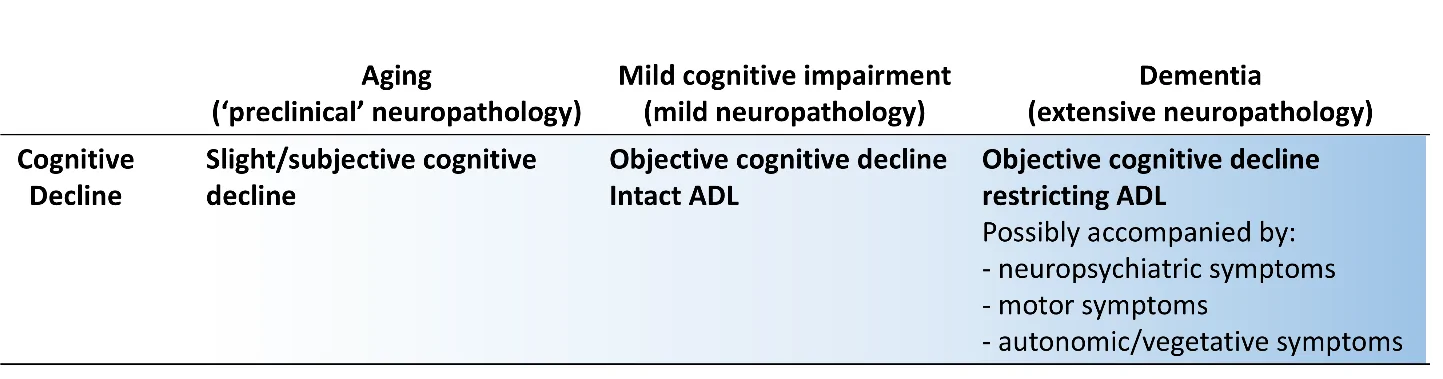
**Traditional view of cognitive decline as primal and pivotal feature leading up to old-age dementia**. Abbreviations: ADL: activities of daily living

Simplified, these views describe a cascade of worsening cognitive functions (Fig. 1). With ageing, people often experience subjective cognitive decline (SCD), not necessarily indicating pathology. If complaints increase and cognitive testing indicates impairments, MCI may be diagnosed. If these impairments restrict ADL, dementia may be diagnosed. Dementia, at its core, is a syndrome of impaired cognition. It may be accompanied by neuropsychiatric symptoms (e.g. apathy, depression, hallucinations), motor symptoms (e.g. gait disturbances), and vegetative/autonomic disturbances (e.g. sleep disorders, orthostatic hypotension).

### Non-cognitive signs

**Figure 2.**
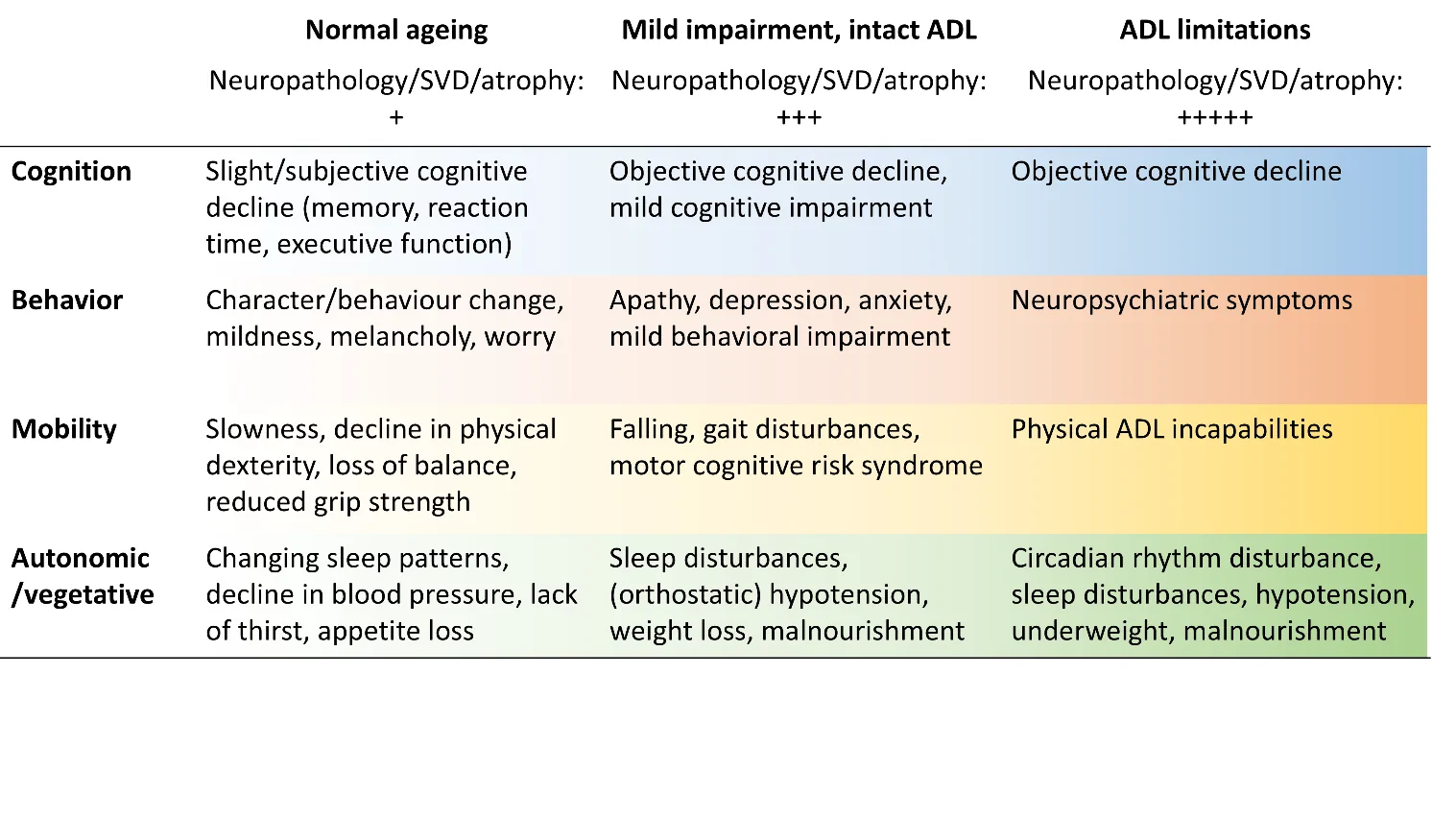
**Hypothesized comprehensive view of cerebral ageing and its associated symptoms ultimately resulting in ADL limitations**. Abbreviations: ADL: activities of daily living, SVD: small vessel disease.

However, next to cognitive symptoms, many symptoms of cerebral dysfunction may precede dementia (Fig. 2). Cognitively healthy older people with mild neuropsychiatric symptoms, including late-life anxiety, depression and apathy have a respectively 45%, 30-50% and 70-90% greater risk of developing dementia than those without [3-6]. Normal mild character changes that may occur with ageing, like worry, melancholia, and diminishing drive, may precede these neuropsychiatric symptoms, similarly to how normal memory complaints may precede MCI.

In this context, the syndrome of “mild behavioural impairment” (MBI) has been defined, comprising changes in behaviour or personality starting later in life, representing a clear change from usual and lasting ≥6 months, unaccounted for by psychiatric conditions or life-events [7]. Although relatively understudied, MBI seems a pervasive precedent of old-age dementia. In a cohort of 2,769 older people without objectifiable cognitive decline, 21% of 743 individuals with isolated MBI developed cognitive impairment or dementia within 3 years, compared to 16% of 254 individuals with isolated SCD, and 31% of 236 with both conditions [7]. This suggests that character changes are at least as potent predictors of future dementia as SCD, despite cognitive impairment being required for a dementia diagnosis.

Next to neuropsychiatric symptoms, motor symptoms in older people, including speed, balance and gait disturbances, are associated with a double to triple incident dementia risk [8]. In this context, the ‘motoric cognitive risk syndrome’ (MCRS) was defined as SCD and slowing of gate speed with intact ADL. MCRS is associated with approximately double dementia risk [9]. Analogous to normal memory decline in the cognitive domain, these motor symptoms also have normal ageing associated precedents including decline in physical speed, dexterity, and grip strength.

Finally, several autonomic/vegetative symptoms and their metabolic consequences precede dementia. For example, cognitively healthy older people with sleep disturbances have a 55-110% higher dementia risk than those without [10,11], and blood pressure, BMI and serum cholesterol decline noticeably 5-15 years preceding diagnosis [12,13]. Hunger, thirst, blood pressure and sleep are all regulated by the hypothalamus, which controls the autonomic nervous system [14]. Although no syndrome has been defined, co-occurring symptoms may indicate dementia risk beyond what would be expected from individual associations, suggesting an overarching phenomenon [15]. These autonomic/vegetative symptoms have ‘normal ageing’ precedents like changing sleep patterns, insensitivity to thirst, and loss of appetite.

No clear causal mechanisms link these late-life neuropsychiatric, motor and autonomic/vegetative symptoms to dementia. Instead, they are likely prodromal. Symptoms mostly predict dementia in the short-term rather than the long-term [5,12,13,16], MBI and MCRS are associated with brain changes that also precede cognitive decline and dementia [9,17-19], and all these disturbances are common features during old-age dementia itself [20].

**Figure 3.**
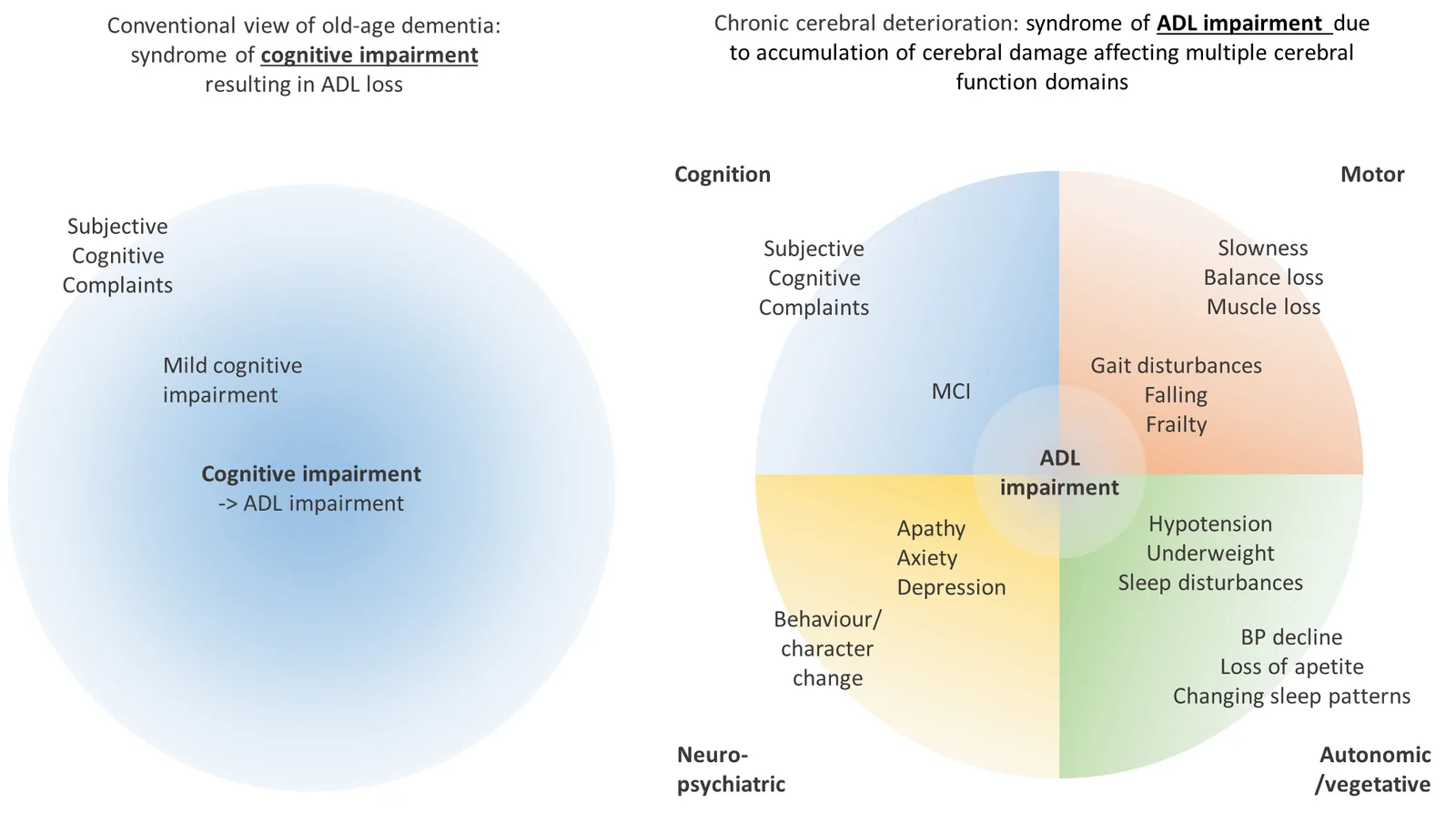
**Traditional view of old-age dementia versus proposed comprehensive, integrated view**. The core of the traditional old-age dementia definition is a syndrome of memory impairment with at least one additional cognitive symptom, leading to ADL impairment. This may be preceded by mild cognitive impairment and subjective cognitive complaints before that. In the proposed integrated view, a syndrome of ADL-impairment is pivotal. This is caused by gradual accumulation of cerebral damage with ageing. In its earliest stages this condition may be preceded by changes in many cerebral functions, including cognition, neuropsychiatric symptoms, mobility symptoms and/or other autonomic/vegetative cerebral functions.

### Chronic cerebral deterioration

This evidence indicates that the current, limited, one-dimensional view of pathological brain changes in older persons leading to SCD, MCI and dementia is incomplete. We hypothesize that gradually accumulating cerebral damage associated with old-age dementia manifests in disturbances across cerebral functions that often precede cognitive dysfunction. Cognitive impairment is merely one pillar of this syndrome. The core symptom is ADL impairment due to gradual cerebral function loss with accumulating cerebral pathology (Fig. 3). This may manifest early in MCI, neuropsychiatric symptoms, cerebral motor impairment, and/or symptoms of vegetative or autonomic dysregulation. Even earlier, it may present in cerebral function changes causing phenotypes commonly associated with normal ageing like mild behavioural and emotional changes, loss of physical dexterity, decreasing appetite, declining weight, declining blood pressure, changing sleep patterns etc. Since dementia refers to the Latin *‘dimenticare’* meaning ‘to forget’, retaining this term is inappropriate because of its limited scope. As an alternative, we suggest *chronic cerebral deterioration* (CCD).

CCD has an acute parallel. Stroke and traumatic brain injury may both acutely cause cognitive symptoms, neuropsychiatric symptoms, motor function loss, and vegetative/autonomic dysregulation. CCD can be seen as its counterpart resulting from gradual accumulation of cerebral damage. Stroke may provide guidance for future study into CCD. Depending on the location and the extent of the cerebral damage, stroke can cause many different symptoms. The various underlying mechanisms, affecting different brain areas, cause varying symptom patterns in stroke patients. However, stroke is the fundamental syndrome, and post-stroke cognitive impairment its cognitive pillar. Analogously, *CCD* is the fundamental syndrome, and old-age cognitive impairment its cognitive pillar.

### Current evidence

The Table 1 presents an overview of potential symptoms pertaining to different non-cognitive CCD domains. The current paper is not an exhaustive systematic review and meta-analysis of all the evidence for the relationships of individual domains and their components with incident dementia. Instead, it refers to highest quality of evidence available, from systematic reviews with meta-analysis, to reviews, to large-scale individual studies. For some of the listed symptoms, the evidence linking them to incident dementia is currently limited. Next to evidence of absence, conceivable explanations for this include the relative lack of attention for non-cognitive symptoms in the context of incident dementia, the scarcity of large-scale long-term longitudinal cohort studies focused on incident dementia that have measured non-cognitive symptoms in cognitively healthy older people, and the lack of well-validated dedicated measurement instruments to quantify some of these symptoms, especially in community-dwelling individuals without dementia in whom early signs may be relatively subtle. Furthermore, not every CCD symptom may be equally predictive of incident dementia, which will depend on the symptom’s measurability, and specificity and sensitivity for CCD.

Evidence for neuropsychiatric symptoms preceding incident dementia is relatively strong, particularly for apathy, anxiety, and depression with late life onset, for which evidence stems from large well-delineated longitudinal cohort studies and systematic reviews with meta-analyses [3-6,16,21,22]. There are a few large longitudinal cohort studies linking psychotic symptoms, agitation and impulsivity with incident dementia, but these areas have been relatively understudied [23-25]. Only a few longitudinal studies have investigated the relationship between personality changes and incident dementia, finding these changes predicted cognitive deterioration [26].

In the motor domain, there is relatively strong evidence from meta-analyses for gait and balance disturbances, reduced mobility, and physical slowness with incident dementia [8,27-29]. Relationships of fine-motor skill, sarcopenia, speaking impairment, chewing and swallowing difficulties with incident dementia have been studied relatively less and associations stem mostly from individual longitudinal cohort studies [8,30-38].

Finally, for the autonomic/vegetative domain possibly linked to thalamic function, there is relatively strong evidence from meta-analyses linking sleep disturbances [39-41], and low/declining BMI [12,13,42], to higher incident dementia risk [39-41]. The evidence from meta-analyses for low and declining blood pressure preceding incident dementia is also relatively strong [12,13,43], although results are not unequivocal [44]. Finally, evidence for hormonal changes [45-49], declining sense of smell [50,51], changes in sex drive [52], generally stems from individual studies or hypothesized based on observations made in patients with dementia, and/or older people.

Future research may systematically review and meta-analyse the evidence for the longitudinal association between non-cognitive symptoms in cognitively healthy older people and subsequent incident dementia, to further substantiate the proposed framework. A complicating factor when discerning between these different prodromal signs of dementia is their potential interrelation. For example, loss of appetite may cause declining BMI, but also lead to metabolic changes like declining cholesterol levels and declining blood pressure, and cause changes in other domains like concentration loss, apathy and sleepiness. Although this may make it difficult to distinguish exactly which symptoms are caused by which underlying mechanism, this underlines that these cerebral symptoms should be studied together, not with an isolated focus on cognition.

**Table 1:** Non-cognitive CCD symptoms and potential precursors in old age.

CCD domain	CCD symptom	Potential precursors in older people
**Neuropsychiatric**	Overall	Personality/behaviour changes, Mild Behavioural Impairment (7,18,24,26,60)
	Apathy	Declining interest in friends, family, or hobbies, social retraction, social isolation, loss of spontaneity, passive conversation, emotional flattening, indifference (5,6,16)
	Anxiety	Worry, restlessness, treatment-resistant late-life onset anxiety symptoms (3,21)
	Depression	Melancholia, fatalism, regarding oneself as a burden, treatment-resistant late-life onset depression (4,21,22)
	Delusions, hallucinations	Indications of (mild) paranoia, untoward suspiciousness of others’ motives, unrealistic self-beliefs, magical thinking (23,24)
	Agitation	Irritability, agitation, argumentative behaviour (24,25)
	Impulse dysregulation	Intrusive behaviour, impatience, impulsivity, recklessness, stubbornness, overeating, neuroticism (24,25)
**Motor**	Overall	Motoric cognitive risk syndrome (8,17)
	Falling, gait disturbances	Loss of balance, changing walking patterns, vestibular function decline (8,27,28)
	Physical slowness, reduced mobility	Declining physical speed, decreasing physical dexterity (8,29)
	Impaired fine-motor skills	Clumsiness, handwriting changes, increasing difficulty with precision tasks in activities of daily living (using utensils, sowing, tying shoelaces) (8,30,31)
	Sarcopenia, frailty	Muscle loss, declining grip strength, increased fatigue (8),(32-34)
	Speaking impairment	Slower talking, stammering, decreasing articulation, changing speech patterns, speaking/language impairment (35)
	Chewing and swallowing difficulties	Swallowing difficulties, subjective difficulty in chewing, oral frailty (36-38)
**Autonomic/vegetative and metabolic consequences:**	Circadian rhythm disturbance, sleeping problems, nocturnal wandering	Changing sleep patterns, daytime sleepiness, napping, difficulty in time estimation, difficulty discerning dreams from real life (39-41)
	(orthostatic) hypotension	Declining blood pressure, orthostatic hypotension (12,13,43)
	Underweight, malnutrition, forgetting to eat, and metabolic consequences	Appetite loss, insensitivity to thirst, declining body weight, declining cholesterol, low on energy, napping (12,13,41,42)
	Changes in sex drive	Changes in sex drive (52)
	Olfactory impairment	Diminishing sense and distinction of smell (50,51)
	Hypothermia	Declining body temperature (61,62)
	Hormonal changes and metabolic consequences (pituitary hormones, cortisol, cholesterol, estrogen, testosterone, thyroid hormones)	Hormonal changes and metabolic consequences (pituitary hormones, cortisol, cholesterol, estrogen, testosterone, thyroid hormones) (45-49,63)

This list is neither conclusive nor exhaustive and meant as a list of (partly purely hypothetical) examples of potential relationships. Note that precursors refer to newly developed symptoms that are uncharacteristic of the person in question and are not due to other life-events. Also note that precursors can overlap and may not be completely confined to single symptom or domain categories. Some precursors may be both prodromal and causal depending on characteristics, e.g. midlife depression may be a causal risk factor, while late-life onset treatment-resistant depression may more likely be a prodromal feature of dementia. References provide context with examples for relationships that have been researched, prioritizing systematic reviews if available.

### Testing the hypothesis

The CCD concept may be tested by longitudinal observational studies in older people, chartering its potential early signs in their relationship to incident dementia. How do symptoms in the cognitive, neuropsychiatric, motor and autonomic/vegetative domains of CCD precede dementia? How do they interrelate? Are their associations more likely to be prodromal than causal? Are individuals with concurrent disturbances in multiple domains more likely to develop incident dementia than expected based on the individual associations for each? These would all provide evidence for CCD’s existence. The hypothesis also predicts new associations with early symptoms that seem unlikely to directly cause dementia themselves. For example, diminished physical dexterity, emotional/character changes, diminished appetite, or hypothermia in healthy older people potentially being associated with increased dementia risk (Table 1). Mapping CCD symptoms to radiological or neuropathological markers of cerebral damage, like atrophy, small vessel disease, and AD proteins, may provide further evidence. Different symptom clusters may involve different locations and/or neuropathology. For example, neuropsychiatric symptoms seem particularly related to cerebrovascular white matter damage and subcortical atrophy, and motoric symptoms more to atrophy and lesions of the premotor and prefrontal cortex [18,19]. Damage may determine the early disease course. For example, relatively isolated accumulation of beta-amyloid and tau may first manifest predominantly as cognitive decline, while frontal, subcortical, and ventricular lesions and atrophy may first manifest with motor and neuropsychiatric symptoms. Strongly mixed pathology may incur symptoms across domains, while relatively isolated pathologies may have relatively distinct longitudinal symptom profiles. These relations can only be uncovered if the early symptoms of CCD are studied across its domains, not with the isolated focus on cognition. Future studies may elaborate in-depth on the biological mechanisms underlying the cognitive and non-cognitive symptom domains of CCD.

To validate the CCD model, ideally large-scale longitudinal cohort studies in older people, preferably without symptoms in the CCD domains at baseline, are needed. Such studies should measure the development and progression of CCD symptoms in relation to ADL symptoms, and incident dementia. When designing new studies, this may require specific development of measurement instruments sensitive to CCD symptoms in early stages, like changes in character and decline of motor function (e.g. decline in speed, dexterity, handwriting). Ideally, studies would include *in vivo* measurements of biomarkers that are also commonly studied in the context of ageing and late-life dementia, like atrophy, (small vessel) cerebrovascular disease, (neuro-)inflammation markers, amyloid, tau, and other late-life dementia related neuropathology [18,53-57]. The gold-standard for biomarkers however remains post-mortem neuropathological assessment. Although not specifically designed for this purpose, many current large-scale longitudinal cohort studies in older people may have (partly) collected the data described above, making the study of the development and progression of CCD symptoms in the context of ageing, ADL decline, and incident dementia possible with readily available data. In future, potential intervention studies aimed at CCD may measure symptoms similarly, although the endpoint would ideally be a diagnosis of CCD according to clinical criteria, not a composite of decline in several measurement scores across CCD domains.

### Implications

CCD provides a more comprehensive understanding of old-age dementia. This complete perspective may help elucidate underlying mechanisms and determinants of disease course. It revises alleged dementia ‘risk factors’ more realistically into prodromal signs of cerebral deterioration; clarifying why risk factors with dubious causal links to dementia, such as apathy, gait disturbance, weight loss, or declining blood pressure, indicate increased dementia risk. CCD provides a clear framework to estimate causality, reducing research waste on dementia prevention unsuccessfully targeting prodromes. CCD is an overarching framework, which can be viewed as replacing previous broader concepts focussed on cognition like ‘dementia’ according to the DSM-V definition. It may overarch more narrowly defined concepts, such as subsyndromes specifically associated with isolated amyloid and tau depositions, or isolated cerebral small vessel disease. However, it covers both the cognitive and non-cognitive spectrum of symptoms that patients may experience in early phases before old-age dementia as it is currently defined.

CCD should not lead to lumping all dementia subtypes together. Within CCD, different aetiologies may induce different chronological symptom patterns. Studying and recognizing these may aid identifying individuals at risk, and developing, targeting, and evaluating future therapies. Thereby facilitating personalized approaches, e.g. based on early symptoms patterns. AD has become a muddled term. It is clinically/colloquially used for typical gradual-onset old-age dementia, while research definitions increasingly abandon symptomatology for AD biomarkers [2], distancing research from clinical practice. This shift in definition towards amyloid away from clinical symptoms may be beneficial for testing and developing drugs specifically targeting amyloid, but may be too narrow for clinical practice [58]. Next to many symptoms in none-cognitive domains, most individuals with old-age dementia show multiple pathologies at autopsy *post-mortem* [59]. Therefore, the current research focus on amyloid and associated memory impairment may only benefit a relatively small proportion of people with old-age dementia. CCD refocuses on symptomatology, leaving room for amyloid-dominant varieties within its concept. Crucially, CCD does not justify medicalisation of individuals without ADL dysfunction. Early signs do not necessarily precede worsening symptoms. Dementia is not normal ageing. However, ageing causes biological wear and tear to all body organs, including the brain, sensitising it to multiple sources of cerebral damage. Rationally, such heterogeneous pathologies in different brain areas cause early noticeable changes in many brain functions.

This manuscript is meant as an initial step towards a broader interdisciplinary initiative to integrate the CCD concept fully into clinical practice. To develop a precisely delineated disease definition that is clinically workable and widely supported throughout the diverse specialties involved with old-age dementia, consensus among clinical and research experts from the relevant fields is paramount. Combining insights from specialties like neurology, psychiatry, gerontology and cognitive science may provide a multidimensional understanding of CCD and the implications of its incorporation into practice and highlight the complexity of old-age CCD. Therefore, the current paper avoids proposing definitive criteria. Provisionally, criteria may be constructed as a reworking of the DSM-V dementia criteria as presented in Box 1, with ADL restriction as the core symptom. All CCD domains can lead to mild ADL difficulties (losing keys, need for cane, homebound because of anxiety), or even ADL restrictions (problems driving). As in current practise for dementia diagnosis according to DSM-V, when exactly ADL difficulties transition into ADL restrictions severe enough to warrant a CCD diagnosis is difficult to determine objectively and may differ between individuals. Because ADL restrictions, or ‘need of care’, are central to the CCD diagnosis, a consensus definition may need to conceptualize this more clearly.

Box 1 Criteria for chronic cerebral deterioration.

**Table:** 

Criteria	Mild chronic cerebral deterioration	Chronic cerebral deterioration
**A**	Decline in ability in everyday activities caused by B without interference in independence (although they may require more time, effort, adaptation, or compensatory strategies)	Interference with independence in everyday activities caused by B.
**B**	Modest gradual changes in one or more cerebral domains (cognitive, neuropsychiatric, motor, or autonomic/vegetative) based on: 1. Concern expressed by individual or reliable informant, or observed by physician 2. Modest impairment documented by objective mental and/or physical assessment	Significant gradual changes in one or more cerebral domains (cognitive, neuropsychiatric, motor, or autonomic/vegetative) based on: 1. Concern expressed by individual or reliable informant, or observed by physician 2. Substantial impairment documented by objective mental and/or physical assessment
**C**	Not exclusively during delirium	
**D**	Not better explained by medication use or other mental disorders.	

Criteria reworked from the dementia criteria according to the Diagnostic and statistical manual of mental disorders, 5th ed.: DSM-5

In general, the potential preventive strategies and interventions that may delay the onset or progression of CCD symptoms would be similar for the ones under study for old-age dementia in general. As in the broader field, the current focus for prevention would be the elimination of risk factors [53]. However, high-risk individuals may be discovered earlier, manifesting non-cognitive symptoms in a stage when cognitive symptoms have not developed. Another difference would be that progression to ADL deficiency may be delayed by offering tailored treatment for specific symptoms, e.g. treatment of neuropsychiatric symptoms may postpone the point at which a patient is reliant on others for their ADL. However, CCD symptoms may be relatively resistant to standard treatment options because of the structural nature of the underlying mechanisms. For example, treatment-resistant late onset depression in late-life seems particularly associated with a higher risk of incident dementia [4,21,22], which may reflect that these late onset depressive symptoms are caused by irreversible structural changes to the brain caused by chronic cerebral damage. CCD may improve counselling of older people with early signs and symptoms when cognitive impairments are still absent. The fact that old-age dementia does not always start with cognitive deterioration but also with other noticeable changes in character, physique, or motor function will concur with the experience of many patients, caregivers, and clinicians. The CCD concept may help them to understand why individual dementia cases may appear so vastly different. CCD may improve access to care. Currently, patients with clear complaints and ADL disturbances may not acquire appropriate care because they do not meet the “cognitive impairment” criterium, leaving doubt about the diagnosis. CCD may provide these patients and their caregivers with adequate explanation and support. Centring the diagnosis around ADL disturbances, emphasizing total cerebral function over cognition alone, CCD focuses on what is clinically the most relevant: the patient’s need of care.

## Data Availability

Only previously published data have been used for this manuscript.
